# Lysine Acetyltransferases and Their Role in AR Signaling and Prostate Cancer

**DOI:** 10.3389/fendo.2022.886594

**Published:** 2022-08-17

**Authors:** Bharti Jaiswal, Akanksha Agarwal, Ashish Gupta

**Affiliations:** ^1^ Integrative Chemical Biology (ICB), Institute for Stem Cell Science and Regenerative Medicine (inStem), Bengaluru, India; ^2^ Epigenetics and Human Disease Laboratory, Centre of Excellence in Epigenetics (CoEE) Department of Life Sciences, Shiv Nadar University, Delhi, UP, India

**Keywords:** androgen receptor, lysine acetyltransferases, prostate cancer, TIP60, P300, PCAF, GCN5, HBO1

## Abstract

The development and growth of a normal prostate gland, as well as its physiological functions, are regulated by the actions of androgens through androgen receptor (AR) signaling which drives multiple cellular processes including transcription, cellular proliferation, and apoptosis in prostate cells. Post-translational regulation of AR plays a vital role in directing its cellular activities *via* modulating its stability, nuclear localization, and transcriptional activity. Among various post-translational modifications (PTMs), acetylation is an essential PTM recognized in AR and is governed by the regulated actions of acetyltransferases and deacetyltransferases. Acetylation of AR has been identified as a critical step for its activation and depending on the site of acetylation, the intracellular dynamics and activity of the AR can be modulated. Various acetyltransferases such as CBP, p300, PCAF, TIP60, and ARD1 that are known to acetylate AR, may directly coactivate the AR transcriptional function or help to recruit additional coactivators to functionally regulate the transcriptional activity of the AR. Aberrant expression of acetyltransferases and their deregulated activities have been found to interfere with AR signaling and play a key role in development and progression of prostatic diseases, including prostate cancer (PCa). In this review, we summarized recent research advances aimed at understanding the role of various lysine acetyltransferases (KATs) in the regulation of AR activity at the level of post-translational modifications in normal prostate physiology, as well as in development and progression of PCa. Considering the critical importance of KATs in modulating AR activity in physiological and patho-physiological context, we further discussed the potential of targeting these enzymes as a therapeutic option to treat AR-related pathology in combination with hormonal therapy.

## Introduction

Prostate cancer (PCa) is one of the most frequently diagnosed solid tumors in men and remains the second leading cause of cancer-related death after lung cancer in men, according to the latest data provided by Cancer Statistics 2022 (1). The prostate is an androgen-dependent organ and the biological effects of androgens are mediated by its binding to the androgen receptor (AR), a well-recognized member of the nuclear receptor (NR) superfamily of ligand-inducible transcription factor (2). Besides being mainly involved in the regulation and development of the male reproductive system and secondary sexual characteristics, androgens/AR also contribute to the physiology of bone, muscle, neural, cardiovascular, immune, and haematopoietic systems (3–5). Deregulated actions of AR signaling has been implicated in the pathogenesis and progression of many AR-related disorders including Kennedy’s disease, androgen insensitivity syndrome (AIS), male infertility, prostatic hypertrophy, and multiple endocrine-related cancers such as prostate, breast, bladder, lung, liver, and kidney (3, 4, 6). In 1941, Charles Huggins and Clarence Hodges first demonstrated the dependence of PCa growth on androgenic hormones and showed that eliminating androgens, either through castration or neutralization of their activity by administration of estrogen, leads to remission of PCa (7). For establishing the relationship between hormones and cancer, and showing the clinical benefits of androgen level manipulation in treatment of prostatic cancer, Dr. Charles B. Huggins was awarded the Nobel Prize in Physiology or Medicine in 1966. The expression of PSA [Prostate-specific antigen or human glandular kallikrein 3 (hK3)], one of the most abundant proteins found in the seminal fluid, is mainly controlled by androgenic hormones and is regulated through the AR at the transcriptional level (8 **)**. It is a serine protease secreted by both normal and neoplastic prostate epithelium and is mainly responsible for liquefaction of the seminal fluid coagulum by proteolytic cleavage of semenogelin I and II, major gel forming proteins present in the semen coagulum. High serum levels of PSA are strongly correlated with advanced stage of PCa and therefore, measurement of PSA levels serve as an important biomarker for clinical detection and evaluation of PCa progression, as well as in the assessment of the patient’s response to PCa drugs. However, several other non-cancerous prostate conditions, such as benign prostatic hyperplasia (BPH) or inflammation or infection of the prostate or recent ejaculation, can also elevate or modify PSA levels, hence, the use of the PSA test alone for diagnosis of PCa is considered controversial by urologists as it can lead to over-diagnosis and over-treatment as part of screening (9). Alternative diagnostic tests such as digital rectal examination, a prostate biopsy, prostate MRI, serum 4K score, and prostate health index (Phi) tests are required for more reliable prediction, diagnosis, and grading of PCa (10).

A wide range of AR antagonists and antiandrogens that block androgen-induced stimulation of the prostate or suppress androgen levels, are frequently used for androgen-dependent PCa therapy (11). In most cases, patients undergoing androgen deprivation therapy show an excellent initial response with complete disappearance of symptoms. However, within a few years therapy fails and the patient develops drug resistance leading to the spread of androgen-independent PCa or castration-resistant PCa (CRPC). Several studies have documented the role of alternative mechanisms, such as amplification of the AR gene, AR overexpression, mutations in the AR gene, variations in CAG repeat length, differential expression of AR splice variants, and abnormal activity of AR-interacting proteins that drive aberrant AR activity and can contribute to the development and progression of CRPC (12, 13). Furthermore, various post-translational modifications (PTMs) may promote abnormal AR activity by direct modification of AR or by modulating the activity of AR interacting proteins and thus, contribute to PCa cell growth, particularly in hormone-resistant PCa (14).

**Figure 1:**
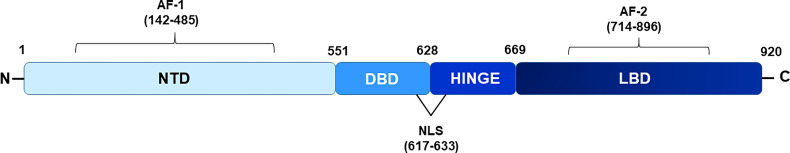
Schematic diagram showing domains of androgen receptor. NTD depicts N-terminal domain, DBD- DNA binding domain, LBD- ligand binding domain respectively. Hinge region connects NTD and DBD. AF-1 and AF-2 (activation function domain 1 and 2) are located within NTD and LBD region respectively. Nuclear localization signal (NLS) is present from 617-633 amino acids.

The AR protein shares common structural features with other nuclear hormone receptors and is comprised of a highly variable N-terminal domain (NTD), a DNA binding domain (DBD), and a ligand binding domain (LBD), each serving specific functions (**Figure 1**). Within AR NTD, a ligand-independent transcriptional activation function (AF-1) domain is present which plays a predominant role in AR-mediated transcriptional activity and is followed by a highly conserved DBD comprised of two cysteine-rich zinc finger-like motifs that facilitate and stabilize AR homodimer binding to androgen response elements (AREs) located within the regulatory region of target genes (4). A short flexible hinge region, located next to the DBD, connects DBD to LBD and contains a bipartite AR nuclear localization signal (NLS) with two clusters of basic residues (^617^RKCYEAGMTLGARKLKKL^634^), crucial for mediating AR translocation into the nucleus (15). Besides playing a significant role in inducing ligand-dependent conformational changes in the AR and regulating subcellular distribution of the AR, hinge region also harbors target sites for various PTMs such as acetylation, methylation, ubiquitylation, and phosphorylation (4, 16, 17). Post-translational modifications in the hinge region can modulate the binding of the AR with its corepressor and coactivator proteins, thereby influencing AR-mediated transactivation function (18). AR LBD folds into 12 α-helices to form a ligand binding pocket and contains a C-terminal ligand-dependent AF-2 domain that serves as a docking site for coactivators or corepressors, depending on the positioning of helix 12/AF-2 relative to the LBD and whether AR is liganded, unliganded, or in an antagonist-bound state (19). AF-2 region facilitates ligand induced direct interaction between the AR-LBD and AR-NTD regions (N/C interactions) which are considered important for full activation of AR (15). In addition, a nuclear export signal (NES) is also located within the LBD and is responsible for exporting AR to the cytoplasm in the absence of ligands (20). The classical mechanism also known as the genomic pathway, by which androgens exerts their action through ARs, involves direct binding of AR to AREs in target genes. In the absence of the ligand, AR is kept in an inactive state in the cytoplasmic compartment, bound by heat shock proteins and co-chaperone proteins (15), however, upon binding to testosterone or dihydrotestosterone (DHT), the two most important endogenous androgens, ARs get freed from the heat shock protein complex and gain active conformation that facilitates its nuclear translocation. Within the nucleus, the AR homodimer binds directly to the AREs, localized within the promoter region of its target genes and recruits coregulators that assist in transcriptional regulation of AR target genes.

## AR coregulators

Like other members of the NR superfamily, transcriptional activity of AR are modulated by recruitment of proteins characterized as coregulators. On the basis of their activating or repressing effect on gene transcription, they are generally divided into two categories that include coactivators, which enhance gene transcription, or corepressors, that suppress gene transcription (21). Coactivators are, structurally and functionally, a diverse group of proteins that frequently target histones and alter chromatin conformation at target genes necessary to allow access of the transcriptional machinery to enable transcription (21, 22). Most NR coactivators contain a conserved helical motif (LXXLL) also referred to as an NR box (nuclear receptor box) that mediates ligand-dependent interactions with different NRs, while an analogous sequence motif (LXXI/HIXXXI/L), termed as the CoRNR box (corepressor nuclear receptor box), can be identified in many corepressor proteins (23). Coregulators can be directly recruited to the chromatin by NR or indirectly as part of large multisubunit coregulator complexes that are subject to dynamic rearrangements determined by the presence or absence of a bound ligand and often act as a scaffold for the assembly and stabilization of additional regulatory proteins (21, 23). Many of them possess specific enzymatic activities such as DNA methyltransferase/demthyltransferase activity, histone acetyl transferase/deacetyltransferase activity, and ATPase dependent chromatin remodeling activity, which help to regulate chromatin architecture and the transcriptional state of the local genomic region (22). Several coregulators of the AR have been reported to selectively fine-tune tissue selective action of AR in various tissues **(** 24 **)**. KATs are an important category of AR coregulator proteins and possess intrinsic acetyltransferase (KAT) activity. KAT-induced acetylation of lysine residues in histone tails neutralizes its positive charge which weakens its interaction with DNA and thus, alters the structure of chromatin and favors the binding of effector proteins to the chromatin, thereby facilitating the process of transcription (25). In addition to acetylating histones, KATs are also known to acetylate non-histone proteins, including NRs, and regulate their activity through various mechanisms such as altering their intracellular localization, expression, stability, ligand-binding affinity, and protein–protein and protein–DNA interactions (26). Consequently, due to their ability to alter the epigenetic landscape, as well as the function of a protein, the deregulated expression and function of KATs play an important role in the development of various human diseases including cancer and therefore, can be targeted for the development of drugs for treating various diseases (27–29). In this review we have discussed the role of various KATs that are involved in modulating AR function and their implications as potential therapeutic targets in PCa treatment.

## Lysine acetyltransferases (KATs) of AR

### p300 (E1A Binding Protein p300 (EP300)/KAT3B)/CBP (Cyclic AMP Response Element (CREB) Binding Protein/KAT3A)

**Figure 2:**
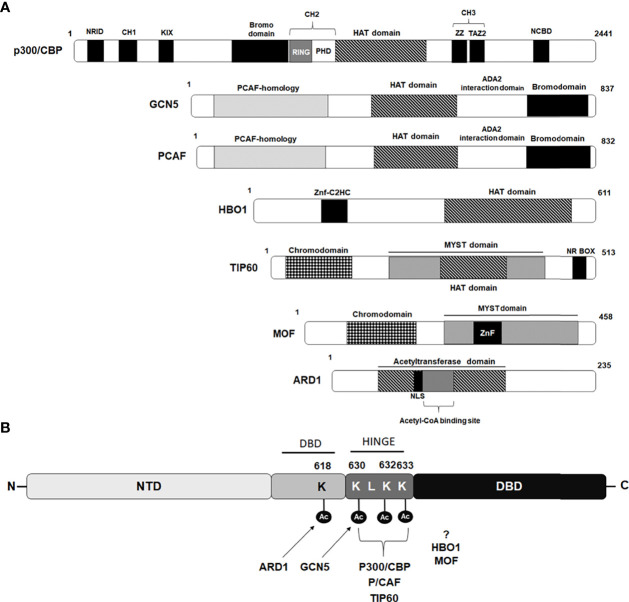
Schematic diagram depicting domains of Lysine acetyl transferases modulating androgen receptor. **(A)** Schematic diagram of lysine acetyl transferases (KATs) depicting their domain organization that interact and modulate androgen receptor activity. **(B)** Amino acids acetylated by different KAT proteins in AR p300/CBP, PCAF and TIP60 acetylate lysine residues 630, 632 and 633 while GCN5 acetylate lysine residue 630 situated within hinge domain, while ARD1 acetylate lysine 618 located in DBD region. Sites targeted for acetylation by HBO1 and MOF are still not been identified.

Both CBP and p300, the two highly homologous lysine acetyltransferase proteins, serve as essential coregulators for many NRs and exert their effect *via* direct acetylation of their substrate proteins at specific lysine residues. In addition to acetylating all four histones and various transcription factors, several NRs, including thyroid hormone receptor (T3R), estrogen receptor (ER), retinoid X receptor (RXR), farnesoid X receptor (FXR), progesterone receptor (PR), androgen receptor, and steroidogenic factor 1 (SF-1) have shown to be direct substrates for p300 lysine acetyltransferase activity (30–39). CBP and p300 share a highly-conserved modular structure comprising of eight distinct domains including a nuclear receptor interacting domain (NRID), three cysteine-histidine-rich regions (CH1, CH2 and CH3), a kinase-inducible CREB interaction region (KIX), acetyllysine-binding bromodomain (BD), and a catalytic lysine acetyltransferase (KAT) domain followed by a nuclear coactivator-binding domain (NCBD) also called as IRF-3 binding domain (IBiD) (39 **)** (**Figure 2A**). The physical interaction of CBP with the AR *in vivo*, and its effect on stimulating AR-mediated transcription, was first demonstrated by Aarnisalo et al. in 1998 (40). In their study, they showed that CBP could competitively inhibit E1A-mediated inhibition of the AR. Furthermore, it has been suggested that CBP plays a role in counteracting reciprocal transcriptional repression of AR and NF-kB by each other, showing multiple roles played by CBP in the AR signaling axis. Subsequently, Fu et al. provided evidence to show AR as a direct substrate for the HAT activity of p300 and further identified that p300-mediated AR acetylation induces DHT-dependent AR transcriptional activity in cultured prostate cancer cells (41).

Additionally, using a combination of mass spectrometry analysis and Edman degradation assays, they identified three lysine residues (630KLKK633) located within the hinge domain of AR, which are targeted for acetylation by p300 (**Figure 2B**). Significantly, they observed that the AR mutant (K632A/K633A mutant) completely abolished AR acetylation *in vivo* and failed to induce ligand-mediated activation of AR on the MMTV promoter in reporter assays (41). Zhong et al. for the first time showed, that p300-mediated acetylation of the AR enhances the stability of the AR protein by inhibiting AR poly-ubiquitination and proteasomal degradation (42). Immunohistochemistry (IHC) performed with human PCa samples revealed that the expression level of p300 protein clearly correlated with the level of the AR protein and that prostate-specific ablation of p300 decreased prostate cancer cell proliferation and tumor growth in the PTEN knockout mouse model, which resulted in an increased survival rate of these mice. Interestingly, these findings suggested an oncogenic function of p300 in prostate cancer in contrast to its recognized role as a tumor suppressor in breast, pancreatic, and endometrial cancers (42). A recent study by Yu and coworkers, studying the 3D structure of ARs with its coactivators by cryoelectron microscopy, revealed that the NTDs of both AR monomers interact with p300 and play an important role in the assembly of the AR-coactivator complex (43). Although p300 and CBP are highly homologous coactivators of AR signaling, the study by Ianculescu showed that 47% of AR target genes depended on p300 for active transcription, whereas only 0.3% were dependent on CBP (44). siRNA-mediated knockdown studies further demonstrated that it is primarily p300 that contributes to androgen-induced H3 and H4 acetylation, H3K4 methylation, TBP and RNA polymerase binding at AR target genes, without affecting AR occupancy (44). Similarly, Jin et al. further uncovered the role of p300 as an important AR co-activator and showed that 83% of androgen-induced direct AR gene targets have overlapping p300 binding peaks located on their promoters/AREs (45). They further found that inhibition of p300/CBP by the small molecule inhibitor, GNE-049 (inhibitor of the CBP/p300 bromodomain), resulted in a significant reduction in H3K27Ac at the AR/p300 overlapping peaks which inhibited AR-mediated transcription, although AR-p300 interaction or binding of AR or p300 to the target gene promoter was not disrupted (45).

The Tindall group worked extensively in the field of PCa to better understand the cellular and molecular mechanisms that drive PCa onset and progression from an androgen-dependent state to an untreatable form, which is mostly androgen-independent. Their work provided substantial evidence to show that under androgen-deprived conditions, the level of the AR coregulator p300 is increased, which can mediate transactivation of the AR by a cytokine, interleukin-6 (IL-6), that facilitate PCa progression *via* the stimulation of proliferation and angiogenesis and inhibition of apoptosis (46–48). More importantly, they found an elevated expression level of p300 detected in immunohistochemistry of PCa patient tissue samples positively correlated with high Gleason score and aggressive prostate tumor types (48). Abnormal morphological alterations in the nuclear structure, including changes in nuclear region, diameter, DNA mass, and DNA index, were additionally reported as a direct effect of high expression levels of p300 in prostate cancer cells (48, 49). Similarly, Comuzzi and coworkers reported that CBP is up-regulated as a consequence of androgen withdrawal in patients with advanced PCa and suggested that under androgen-deprived conditions, CBP can mediate aberrant AR activity which could explain the failure of endocrine therapy in late-stage PCa (50). Clinical investigations have reported significantly increased levels of IL-4, another cytokine, in the serum of androgen-independent PCa patients that leads to transactivation of PSA (51). Later, it was recognized that IL-4 induces the expression of p300/CBP, their interaction with AR, p300/CBP-mediated acetylation of AR and the recruitment of AR-p300/CBP complex onto AREs, leading to unsolicited activation of AR signaling in the absence of androgens (52). Altogether these findings provide evidence to show that p300 plays a prominent role as an AR coactivator modulating the local chromatin environment that facilitates the regulation of AR transcriptional activity. Furthermore, these findings provide evidence to show that ligand-mediated induction of AR activity can be modulated by acetylation of AR at critical lysine residues and the presence of multiple lysine sites that can be acetylated by single acetyltransferase, points to a regulatory role of acetylation in stimulation of AR activity.

### PCAF (p300/CBP-Associated Factor)

PCAF was identified as a p300/CBP interacting protein and belongs to the superfamily of GCN-5 related N-acetyltransferases (GNATs) (53). The PCAF protein consists of 832 amino acid residues and is characterized by the presence of an N-terminal domain that interacts with transcriptional activators followed by a highly-conserved HAT domain and a bromodomain that can interact with acetylated lysine (**Figure 2A**). PCAF is one of the two paralog proteins found in mammals that are homologous to the yeast GCN5 and in contrast to p300 and CBP paralogs that show high redundancy and are largely interchangeable in their functions, PCAF and GCN5 knockout studies in mice demonstrated only partial redundancy in their functions (54, 55). PCAF forms an important component of two multisubunit mammalian HAT complexes, STAGA (SPT3-TAF9-GCN5/PCAF acetylase), and ATAC (ADA two A containing/complex), and have the ability to influence various cellular processes including regulation of chromatin structure and gene transcription *via* acetylation and modification of nucleosomes in cooperation with other proteins (56). Direct interaction of PCAF has been reported with several NRs such as glucocorticoid receptor, estrogen receptor α, and retinoic acid receptor, where PCAF has been shown to act as a transcription co-activator for these NRs (57, 58). PCAF is a well-known co-activator of AR and can regulate AR activity by directly interacting with AR and acetylating at lysine residues 632 and 633, similar sites acetylated by p300 (**Figure 2B** **)** (41). Fu and coworkers showed that the N-terminal domain and the carboxyl terminus, including the bromo domain of PCAF, is required for full binding to the AR (41). Interestingly, the study by Gong et al. found upregulated levels of PCAF in human PCa cell lines and showed that elevated levels of PCAF enhanced ligand-dependent AR transcriptional activity (59). An inverse correlation was found between the expression levels of PCAF and micro RNA, miR-17-5p. Consequently, downregulated expression level of miR-17-5p promoted unsolicited activation of the AR in PCa cells through heightened PCAF expression (59). A recent study by Zhou et al., showed that PCAF can acetylate delta-catenin and cause its degradation through autophagy by the Atg5/LC3 pathway. By degrading delta-catenin, PCAF can suppress PCa cell growth in both AR-positive and AR-negative PCa cells (60). Altogether these findings suggest that PCAF can contribute to both AR-dependent and AR-independent functions in PCa cells.

### GCN5 (General Control Non-repressed 5 Protein)

GCN5 or KAT2A that belongs to the GNATs family of HAT is a highly-conserved lysine acetyltransferase shown to be present in humans and other organisms including mice, zebrafish, yeast, and plants (61). Human GCN5 is an 837 amino acid long protein with presence of an extended amino-terminal domain or PCAF homology domain, a conserved HAT domain, an ADA2 domain, and a bromodomain (**Figure 2A**). GCN5 shares more than 70% identity with PCAF and like PCAF, GCN5 exists as a catalytic component of two multiprotein complexes, SAGA (subunit of the Spt-Ada-Gcn5-acetyltransferase) complex and ATAC (Ada Two A-containing) complex and regulates target gene expression by preferentially acetylating lysine 14 on histone H3 (H3K14ac) and lysine 8 and 16 on H4 (H4K8ac and H4K16ac) (62–65). Indeed, the effect of acetylation by any HAT protein as an inducer of transcription was shown for the first time using only this HAT protein (66). Like various KAT proteins, GCN5 can catalyze acetylation on both histone and non-histone proteins and thus contributes to a range of cellular functions including gene transcription, chromatin organization, cell growth and differentiation, autophagy, neuronal apoptosis, stem cell differentiation, and hematopoiesis (61, 67, 68). Genetic studies have revealed an important role for GCN5 in normal embryonic development, as mice embryos homozygous null for GCN5 (*Gcn5-/-*) die as early as day 10.5 post-coitum (69). GCN5 serves as an oncoprotein in various cancer types and upregulated expression of GCN5 has been reported to promote hepatic cancer, breast cancer, lung cancer, and colon cancer and is often associated with aggressive tumor progression and poor prognosis (61, 70). Conversely, GCN5 depletion or inhibition of its HAT activity has been shown to induce apoptosis and reduce viability of Burkitt lymphoma cells as well as lead to increased sensitivity of ER-positive breast cancer cells for tamoxifen (65, 71).

Wang and coworkers, demonstrated for the first time, the role of GCN5 in inducing ligand-dependent AR-mediated transactivation at subset of the AR target genes such as PSA, vinculin, and p21 (72). They showed that the recruitment of GCN5 to cis-regulatory elements of AR target genes is facilitated through the MDC1 protein (mediator of DNA damage checkpoint protein 1) acting as a scaffold and together, this coactivator complex induces AR function by increasing histone H3 acetylation level on cis-regulatory elements (72). Interestingly, in a study conducted by Shao et al. demonstrated that IL-6 induces GCN5 expression in PCa cells and GCN5 knockdown hinders PCa proliferation, migration, invasion, and EMT thereby, promoting IL-6 stimulated metastasis of PCa. By further dissecting the mechanism of GCN5, they could show that GCN5 modulate the PI3K/PTEN/AKT signaling pathway (73). Since IL6 is a well-known androgen-independent AR transcriptional activator and this study shed light on IL6-induced GCN5 expression in PCa cells. However, whether since IL-6-induced GCN5 had any role in regulating AR intracellular dynamics or AR downstream signaling, remains unexplored in this study. Recently, Ding et al. has shown that lysine 620 acetylation of LIFR (leukemia inhibitory factor receptor) correlates with PCa progression. They further showed that GCN5-mediated acetylation of LIFR at 620 leads to its dimerization and AKT activation, that consequently promotes its downstream signaling, leading to PCa progression (74). They also showed that AKT in a feedback loop can stabilize GCN5 by inhibiting GSK3-β mediated degradation of GCN5. Contrary to this evidence of GCN5 being oncogenic in PCa, a recent study by Ghildiyal et al. showed that GCN5 binds on upstream region of lncRNA NXTAR (positioned subsequent to the AR gene) and enhance H3K14ac marking leading to enhanced expression of NXTAR upon ACK1 inhibition (75). NXTAR expression epigenetically silences AR gene expression and also reduces enzalutamide-resistant prostate tumor growth. These studies exhibit contrasting functions of GCN5 where it acts as coactivator of the AR for AR-targeted gene transcription but can also suppress the AR synthesis by suppressing AR gene transcription. Collectively these findings reveal conflicting information about GCN5 in prostate cancer, with GCN5 being shown to be a cancer-inhibiting as well as a cancer-promoting factor in the case of PCa and suggests GNN5 might play a context-dependent role in PCa. Recently, Lu and coworkers demonstrated that GCN5/KAT2A can acetylate AR at the K630 residue and induce its translocation into the nucleus (76) (**Figure 2B**). Acetylated AR, in turn, further induced the expression of KAT2A in a positive feedback loop manner. The elevated expression of KAT2A in PCa tissues was found to be significantly correlated with poor clinical outcomes in PCa patients and also promoted resistance to arbiterone treatment in CRPC abiraterone therapy in CRPC. Overall, these findings suggest KAT2A/GCN5 as a novel therapeutic target and inhibiting GCN5 activity can be particularly useful for treatment of abiraterone-resistant CRPC **(** 76).

## TIP60 (HIV-Tat interactive protein 60kDa/KAT5)

TIP60, a member of MYST histone acetyltransferase family, is characterized by the presence of a chromodomain, a highly conserved catalytic MYST domain composed of an acetyl-CoA binding motif, a zinc finger motif, and an NR Box motif located at C-terminus (**Figure 2A**). The HAT activity of TIP60 was first identified by Yamamoto and Horikoshi, where they demonstrated that TIP60 preferentially acetylates lysine residues on the amino-terminal peptides of histones H2A, H3, and H4 (77). Subsequent studies showed that TIP60 is an important part of the multisubunit complex of 18 proteins, which have been found to possess other enzymatic properties including HAT, ATPase, DNA helicase, and DNA binding activities (78, 79). TIP60 has been shown to have a role in many important biological processes, including DNA damage repair, chromatin remodeling, transcriptional regulation, cell cycle progression, apoptosis, wound healing, and nuclear receptor signaling (79–81). In addition to acetylating histones, TIP60 can also acetylate a wide range of non-histone target substrates, including p53, ATM, c-Myc, STAT3, NF-κB, CREB, E2F1 and many nuclear receptors (79, 82). Genetic studies in mice revealed TIP60 to be essential for normal development and survival, as homozygous TIP60 knockout mice embryos do not survive beyond the blastocyst stage of development (83). The earliest evidence showing a role for TIP60 in modulating NR signaling was provided by Robson and coworkers, where they demonstrated that TIP60 acts as a transcriptional coactivator for various class I NRs such as androgen, estrogen, and progesterone receptors (84, 85). They also showed that TIP60 interacts directly with AR through its NR box (nuclear receptor box; LXXLL) motif and that this interaction is not dependent on AR ligands, thereby highlighting the importance of TIP60 in modulating AR functions. Subsequently, the study by Shiota et al. showed that TIP60 interacts with AR and acetylate three lysine residues (lysine 630, 632, and 633) located in its hinge region (**Figure 2B**) (86). TIP60-AR interaction and TIP60-dependent AR acetylation were shown to promote AR nuclear localization as the knockdown of TIP60 reduced AR accumulation in the nucleus. TIP60 was also shown to bind to the PSA promoter in both a ligand-dependent and ligand-independent manner and promote cell proliferation of both androgen-sensitive and androgen-insensitive PCa cells (86, 87). Since TIP60 can act as a coactivator of AR, even in the absence of its ligand, this highlights its role in both PCa and CRPC, acting either through the AR receptor signaling axis as well as through unknown pathways. Given its involvement in modulating AR signaling and prostate disease pathology, TIP60 has emerged as a potentially useful but challenging therapeutic target.

## MOF (males-absent on the first)

MOF, also known as KAT8 or MYST1, is a well-recognized lysine acetyltransferase member of the MYST family. It was initially identified in Drosophila, as one of the critical component of the X chromosome dosage compensation complex that is required to maintain equal expression of X-chromosome-related genes between different biological sexes (88, 89). The structure of MOF is closely related to TIP60 and shows the presence of a N-terminal chromo-like domain located adjacent to the catalytic MYST domain that includes an acetyl−CoA binding domain and C2HC−type zinc finger domain (**Figure 2A**) (90). In humans, MOF is shown to be a part of two different multiprotein complexes, MSL and NSL, both of which can acetylate H4K16 (91). Besides dosage compensation, the role of MOF has been identified in a wide range of cellular functions including DNA damage repair, cell cycle, gene expression regulation, maintenance of chromatin architecture, embryogenesis, and ESC self-renewal (91–93). A genetic study in mice showed that the embryo of KAT8 null mutant mice dies very early at the preimplantation stage, suggesting the critical function of KAT8 in normal embryonic development and maintenance (92, 93). Although MOF is closely related to TIP60, they do not show much functional overlap and display limited redundancy for some functions. For instance, during DNA damage, both MOF and TIP60 can play a similar role in deciding the fate of the cell by either pushing it toward cell cycle arrest or apoptosis by acetylating p53 on lysine 120 (94). Several studies have reported significant downregulation of MOF and associated decrease in the acetylation level of H4K16 in variety of human cancers including primary breast carcinoma, medulloblastoma, ovarian epithelial cancer, colorectal carcinoma, gastric cancer, and renal cell carcinoma. Also, upregulated levels of MOF have been reported in non-small-cell lung cancer and in glioblastoma (GBM), the most common and lethal malignant tumors of the central nervous system (91, 95–97).

Jaganathan et al. identified direct interaction of MOF with the AR by immunoprecipitation assay in LNCaP cells. They further showed that there is competitive binding between the AR and SIRT1 with MOF. MOF is shown to act as coactivator of the AR as it can enhance promoter activation in a reporter plasmid assay and by acting as a transcriptional co-activator of the AR, MOF can regulate genes for cell proliferation, cell cycle, and apoptosis in PCa cells (98). However, in this study, no direct acetylation of the AR was shown by MOF. Interestingly, they also showed that MYST can augment NF-κB transcriptional activity in the presence of the AR. In a subsequent study, Kim et al. showed that MOF is recruited on AR targeted gene promoters and can lead to their transcriptional activation in a ligand-dependent manner (99). The binding of MOF on AR targeted genes was facilitated by WDR5 (tryptophan, aspartic acid repeat containing protein 5) leading to increased marking of H4K16ac on these promoters, which resulted in the transcriptional activation of AR targeted genes such as KLK3, TMPRSS2, FKBPS, and IGFIR (99). Interestingly, the observation that the reduced level of MOF in AR negative PCa cells leads to apoptosis sheds light on, hitherto, unknown functions of MOF in PCa in both the presence and absence of the AR, making it an attractive molecular target for CRPC treatment.

## HBO1 (Histone acetyltransferase binding to ORC1)

HBO1 (also called as KAT7 or MYST2), a member of the MYST family of lysine acetyltransferases, was originally identified as an interacting partner of ORC1, the largest subunit of the origin recognition complex, and like other members of MYST family, HBO1 contains a highly-conserved catalytic MYST domain and a unique N-terminal serine rich domain (100) (**Figure 2A**). HBO1 exists as a part of multiprotein histone acetyltransferase complex and preferentially acetylate histone H4 along with H3 and H2A (100, 101). Although it was initially recognized for its role in DNA replication licensing, HBO was later shown to participate in the transcriptional regulation of patterning genes essential for postgastrulation embryonic development in mice, as absence of HBO1 caused drastic reduction in expression of many genes required for normal embryonic development, potentially as a consequence of significantly reduced histone 3 lysine 14 (H3K14) acetylation mediated through HBO1 (102). In a recent study, Yang et al. showed the physiological significance of HBO1 in regulating hematopoietic stem cell maintenance and self-renewal through histone acetylation and the transcription of HSC genes (103).

Sharma et al. demonstrated, for the first time, direct interaction of HBO1 with the AR using a yeast two hybrid system, pull-down and immunoprecipitation assays (104). Androgen independent interaction of the AR with HBO1 was shown to be mediated by the AR DBD and LBD region and HBO1 zinc finger and the acetyltransferase domain, however, whether HBO1 could directly acetylate the AR was not examined in the study. Remarkably, unlike other HAT proteins, which normally function as a coactivator of AR, HBO1 interaction with the AR was shown to inhibit AR-mediated transcription, suggesting HBO1 functioning as a corepressor for AR, however the mechanism of HBO1-mediated repression of AR activity remained unidentified (104). Notably, HBO1 is one of the most abundant lysine acetyl transferase expressed in normal tissues in humans, with highest expression detected in the testis and ovary (105). Expression analysis of HBO1 and the AR during testis development showed an age-related increase in expression of both the proteins (106). Elevated expression of HBO1 has also been reported in many cancers including testis, ovarian, breast, bladder, gastric, and prostate (101, 105). Whether the abnormal expression and gene amplification of HBO1 reported in PCa contributes in any way to the development or progression of PCa is an interesting topic to explore (105). Interestingly, of all known lysine acetyltransferases that interact with the AR, HBO1 has only been shown to repress the transcriptional activity of the AR and can modulate the selective expression of the AR target genes. Additionally, high expression of HBO1 has been found exclusively in samples of normal testis and prostate tissue, although the precise physiological and pathophysiological functions of HBO1 in the prostate, as well as the physiological consequence of HBO1-mediated suppression of AR transcriptional activity, are not fully understood.

## ARD1 (Arrest-defective 1 protein)

Also known as N-α-acetyltransferase 10 (NAA10), is an N-acetyltransferase (NAT) enzyme that belongs to the GCN5-related N-acetyltransfererase (GNAT) superfamily that is widely-known to catalyze the transfer of an acetyl group from acetyl coenzyme A to the target protein’s N-terminal amino acid (107). Although ARD1 is best known for its role as a NAT, accumulating evidence has revealed that ARD1 also exhibits KAT activity and can acetylate many proteins including HIF-1α, β-catenin, myosin light chain kinase (MLCK), runt-related transcription factor 2 (Runx2), phosphoglycerate kinase 1 (PGK1), heat shock protein 70 (Hsp70), and androgen receptor (107, 108 **).** Important roles for ARD1 have been detected in a wide variety of cellular activities like cell division, proliferation, hypoxia, apoptosis, and autophagy (107). Not surprisingly, researchers have found the aberrant expression and deregulated KAT activity of ARD1 contributes to different cancers including lung, liver, breast, cervical, bladder, and colorectal cancers (107, 109, 110). The human ARD1 protein consists of 235 amino acid residues with a well-conserved N-acetyltransferase domain that contains an acetyl-CoA-binding site, a putative nuclear localization signal (NLS), and a highly variable C-terminal region, recognized in different ARD1 variants derived from alternative RNA splicing (**Figure 2A**) (111, 112). Like other AR coregulators, various studies have reported elevated expression levels of ARD1 protein in lung, liver, breast, cervical, bladder, and colorectal cancers (113–116, 117–119). Wang et al. revealed a role for ARD1 as an oncoprotein in PCa rather than a tumor suppressor, as described earlier by Kuo et al. in breast cancer (117, 120). The first evidence to show implication of ARD1 in the regulation of AR-mediated transcriptional function and in development of prostate cancer was reported by Wang and coworkers in 2012 (120). They showed that direct interaction of ARD1 with AR facilitated the binding of the AR to its targeted gene promoters and demonstrated that ARD-1 dependent acetylation of the AR enhances AR-mediated gene transcription and tumorigenesis of prostate cancer cells (120). Lysine 618 (K618) residue located within the DBD region was identified to be the primary target site in AR acetylated by ARD1 (121). Interestingly, the site targeted by ARD1 differs from the sites targeted by other lysine acetyltransferases like TIP60 and p300, which are situated in the hinge region of the AR. Moreover, ARD1-mediated AR acetylation at K618 was found to induce nuclear translocation of the AR in a ligand-dependent manner and knockdown of ARD1 was shown to dramatically inhibit nuclear import of the AR in LNCaP cells (109, 121). To identify the mechanism of ARD1-mediated AR nuclear translocation, using sequential coimmunoprecipitation analyses, DePaolo et al. further identified that ARD1 forms a ternary complex with the AR and HSP90 in cytoplasm. However, in the presence of androgen, the expression level of the ARD1 protein, as well as ARD1-mediated acetylation of AR, significantly increases and facilitates the dissociation of acetylated AR from HSP90 in a dose-dependent manner leading to rapid translocation of acetylated AR into the nucleus (121). The consistently upregulated expression level of ARD1 protein detected in human PCa cell lines and prostate tumor tissue samples has been suggested to promote prostate tumorigenesis through a positive-feedback mechanism, where ARD1 gets upregulated and functionally activated by androgen in an AR-dependent manner and, in turn, positively regulates AR activity by interacting with AR and acetylating it to promote AR nuclear translocation (120, 121). Since, a high ARD1 expression level was shown to promote proliferation of PCa cells and tumor growth, measurement of the ARD1 expression level was suggested to serve as an important marker for determination of prostate cancer (120). These findings suggested ARD1 as a potential therapeutic target for development of small molecule inhibitors for PCa therapy and targeting ARD1-mediated AR acetylation and AR-HSP90 dissociation as an alternative strategy to regulate AR activity for prostate cancer therapy.

## Targeting KATs for prostate cancer therapy

Although there is no definitive cure available for advanced and metastatic prostate cancer, several therapeutic options are available that can prolong survival and improve the patient’s quality of life for quite some time. A number of factors, such as the patient’s age, health, type of symptoms, PSA level, grade of tumor, extent of tumor spread, and response to the therapy, can help in making treatment plan decisions and predicting outcomes. As over 80% of PCa shows dependence on androgens, most treatments are directed towards inhibiting the AR activity either by using antiandrogens, lowering the secretion of testosterone by surgical castration, or by using luteinizing hormone-releasing hormone (LHRH) agonists and antagonists (122, 123). Cyproterone acetate (CPA), a highly potent steroidal anti-androgen was one of the first drugs approved for the treatment of PCa that acts by competing with endogenous AR ligands, DHT, and testosterone for binding to the AR (124). However, its use has been limited due to several undesired observed side effects such as gynecomastia, sexual dysfunction, fatigue, weakness, bone density loss, and reversible infertility in patients undergoing CPA treatment (**Table 1**). Currently, CPA is not approved by the FDA for use in the United States but is still widely used in many other countries. Several clinically effective non-steroidal antiandrogens such as flutamide, bicalutamide, and nilutamide were subsequently introduced with several advantages over steroidal antiandrogen for PCa treatment (124, 125). In contrast to nilutamide and flutamide, bicalutamide (casodex) has a better safety and tolerability profile and is the most potent widely-used first-generation antiandrogen (**Table 1**) typically given with LHRH agonists (e.g., goserelin or leuprolide) to treat stage D2 metastatic prostate cancer. The search for more efficacious and potent antiandrogens led to the development of next-generation antiandrogens that includes abiraterone acetate, enzalutamide, and recently approved apalutamide and darolutamide (**Table 1**). Enzalutamide, a popular antiandrogen drug, inhibits the growth of castrate-resistant prostate tumors, not only by blocking androgen binding to the AR but also by obstructing AR nuclear translocation, DNA binding, and co-activator recruitment (130). Except for darolutamide, both enzalutamide and apalutamide can penetrate the blood–brain barrier (BBB) and results in central nervous system side effects, including increased risk of seizures due to their binding to and inhibition of the γ-aminobutyric acid receptor (**Table 1**).

**Table 1:** List of drugs used in management of prostate cancer.

Type of Drugs	Compound	Target	Applications	Common adverse effects	Ref.
**Androgen Receptor blockers**					
**Steroidal anti androgen** (synthetic derivatives of hydroxyprogesterone)	Cyproterone acetate (CPA) Megestrol acetate Medroxyprogesterone acetate	Competitively block testosterone and its active metabolite from binding to the androgen receptor relatively low affinity to the AR. show a partial agonist effect	Used for treating advanced PC	Hot flashes, loss of libido and erectile dysfunction, nausea, diarrhea, cardiovascular and hepatotoxicity	(124, 125)
First generation non-steroidal antiandrogen (derivatives of anilide)	Flutemide Nilutamide	Competitively inhibit the binding of testosterone and DHT to the AR Exert pure AR antagonistic activity	Used at a higher dosage as a monotherapy without castration Used for treatment of metastatic (stage D2) prostate cancer, together with a luteinizing hormone-releasing hormone (LHRH) analog or surgical removal of the testicles to treat advanced prostate cancer	Gynecomastia,hot flashes, diarrhea, anemia breast pain, elevated liver, transaminases, hepatic toxicity, gastrointestinal toxicity	(124, 125)
Second generation antiandrogens	Bicalutamide (Casodex)	Higher affinity for AR, than flutamide and nilutamide	Can be used as monotherapy at higher dosage Used for treatment of metastatic (stage D2) prostate cancer, together with a luteinizing hormone releasing hormone (LHRH) analog or surgical removal of the testicles to treat advanced prostate cancer	Gynecomastia, hot flashes, decreased libido, impotence, breast pain, hepatic toxicity,gastrointestinal toxicity	(124, 125)
Third generation antiandrogens	Enzalutamide Apalutamide Darolutamide	Exclusive AR antagonist higher binding affinity for the AR compared with bicalutamide Block nuclear translocation of AR Inhibit DNA binding of AR Inhibit recruitment of AR cofactors	Used for the treatment of metastatic hormone sensitive PCa(mHSPC) Used for non-metastatic CRPC (nmCRPC)	Fatigue, back pain, hot flashes, hypertension, constipation, headache No risk of seizures reported with Darolutamide till date	(124–126)
Androgen biosynthesis inhibitors	Arberaterone acetate Ketoconazole	Blocks the action of cytochrome P450 enzyme 17r-hydroxylase-17,20-lyase (CYP17A1) in the testes and the adrenal glands inhibit production of DHT and decrease endogenous androgen levels.	Used for metastatic CRPC treatment and high-risk castration sensitive Pca Used for metastatic CRPC	Hypokalemia,fluid retention, and hypertension, hepatotoxicity, adrenocortical insufficiency Motor neuropathy and ototoxicity, hepatotoxicity	(126, 127)
**Luteinizing hormone releasing hormone (LHRH) analog**					
LHRH agonists	Leuprolide acetate Goserelin Triptorelin	Chronic exposure to LHRH agonists results in the down-regulation of LHRH-receptors, suppressing LH and FSH secretion This lowers testosterone levels to castrate levels.	To treat stage D2 metastatic prostate cancer	Bone loss, decreased libido, ED, weight gain, cardiovascular problems. Testosterone surge after initial injection of LHRH agonists can result in flare of prostate cancer symptoms in majority of patients	(128, 129)
LHRH antagonists	Degarelix Relugolix	Immediately bind to LHRH receptors, causing instant decrease in LH, FSH and testosterone levels without any flare	Advanced hormone-dependent prostate cancer	Injection-sitereaction, hot flushes, reduced libido and erectile dysfunction	(128, 129)

AR inhibitors do not prevent androgen from being produced in the body, therefore, they are preferably used in combination with orchiectomy (testicular castration) or an LHRH (gonadotropin-releasing hormone (GnRH)) analogs to achieve combined androgen blockade or total androgen blockade (131). Although androgens are secreted mainly in the testes under the control of luteinizing hormone (LH), a significant amount of androgens is also produced by the adrenal glands (132). When hypothalamus sense the low levels of circulating testosterone in the blood, it releases GnRH that induces the anterior pituitary gland to secrete LH, while higher levels of circulating testosterone causes the hypothalamus to stop releasing GnRH (132). Continuous treatment with LHRH agonists overstimulate GnRH receptor down-regulation which prevents the pituitary gland from secreting LH, thus lowering the level of androgens in the body (128 **)**. Indeed, treatment with LHRH agonists are also used as a preferred alternative to surgical castration. Leuprolide, goserelin, and triptorelin are clinically approved LHRH agonists to treat PCa, but can cause testosterone flare as a noted side effect observed during the first few weeks of treatment which is usually countered by coadministration of antiandrogen drugs (133). Whereas the LHRH antagonists (degarelix and relugolix) block the pituitary LHRH receptor suppressing the release of gonadotropins and testosterone more quickly without causing the testosterone flare (**Table 1**).

Despite compelling clinical advantages, long-term use of all classes of PCa drugs can unfortunately cause adverse complications ranging from decreased muscle mass, reduced bone density, erectile dysfunction, anemia, increased body fat, cardiovascular events, and hot flashes, partly due to widespread and non-specific activation/inhibition of the AR in many different tissues (**Table 1**). Also, a large majority of patients develop resistance as the treatment proceeds and eventually stop responding to these drugs causing PCa recurrence. To counter these challenges, another category of chemically synthesized compounds called selective androgen receptor modulators (SARMs) were introduced and, depending on their chemical structure, differentially bind to the AR as agonists, antagonists, partial agonists, or partial antagonists and can have varied effects within different tissues due to variable cofactor recruitment to facilitate tissue-specific benefits (134). These were initially developed as a substitute for steroidal androgens to treat osteoporosis, partial androgen insensitivity syndrome (PAIS), benign prostatic hyperplasia (BPH), cachexia, and certain muscular dystrophies, however, due to their ability to target the AR function more specifically in certain tissues with minimal off-target effects, clinical applications of SARMs are being explored as a prospective treatment for PCa, particularly in managing symptoms like decreased muscle mass and reduced bone density in patients undergoing hormonal therapy for PCa (134). Similarly, AZD3514 (AstraZeneca, Macclesfield, UK), a selective androgen receptor down-regulator (SARD), has also been tested in CRPC, due to its ability to inhibit ligand-mediated AR signaling *via* obstructing AR nuclear translocation, downregulating the AR levels, and inhibiting the expression of AR-dependent genes such as PSA and TMPRSS2 (135). However, this compound has been shown to have only moderate antitumor activity in CRPC patients with low tolerability and associated toxicities such as nausea and vomiting (136).

Several studies have reported altered expression levels and deregulated activities of AR coactivator proteins as critical contributors in maintaining anomalous AR activity that drive prostate tumor growth even at castrate levels of testosterone, particularly in development of CRPC and thus, have generated a great deal of interest in targeting these coregulators for PCa therapy (48, 50, 137–141). Recently, Liu et al., performed a systematic analysis to determine the individual contribution of several coregulators, including KAT proteins (p300 and TIP60), in the regulation of AR activation with relevance to PCa initiation and progression (141). They investigated the role of 18 AR coregulators and showed that coregulators confer selectivity in AR action in a gene-specific manner, highlighting the dependence of AR signaling on its coregulators, including lysine acetyltransferases such as TIP60 and p300. Several laboratories have designed and developed small molecule inhibitors to abolish or control AR activity by targeting various AR coactivators. Santer et al. investigated the anti-prostate cancer effect of C646, a small molecular inhibitor of p300/CBP catalytic activity, and found that inhibition of p300 catalytic activity leads to induction of caspase-dependent apoptosis and the reduction of cellular proliferation in several androgen-dependent and castration-resistant PCa cells, triggered by a substantiated decrease in AR function and impaired NF-κB signaling through p65 degradation (142). Tohyama and coworkers identified two strictly specific inhibitors of p300 catalytic activity, NK13650A and NK13650B (with an IC_50_ values of 11 nM and 22 nM, respectively), which were obtained as secondary metabolites by culturing a production strain belonging to *Penicillium*sp. and examined their effect on the viability of DHT-dependent LNCaP cell line and DHT-independent PC-3 cell line (143). Interestingly, they found that treatment with NK13650s suppressed ligand-induced AR transcriptional activation and augmented the death of both hormone-dependent and hormone-independent growth of prostate cancer cells suggesting NK13650s as potential new lead compounds for the treatment of androgen-independent PCa. Despite effectively inhibiting p300 catalytic activity at low concentrations, the particularly high concentration requirements of these inhibitors were a significant drawback to demonstrate their growth inhibitory effect against these cancer cells. Chemical modification was suggested as a possible way to enhance the efficacy and incorporate drug-like properties to these inhibitors for clinical testing purposes. In the search for identifying specific and potent inhibitors of p300 catalytic activity for the purpose of inhibiting both androgen sensitive and castrate resistant PCa growth, Lasko and coworkers conducted virtual screening of approximately 800,000 compounds and identified a highly selective p300/CBP catalytic inhibitor, A-485, that was at least 1000-fold more potent than previously reported p300 inhibitor C646 and showed favorable ADME properties and PK profile (144). Notably, A-485 treatment could inhibit DHT-stimulated PSA expression more potently than the AR antagonist enzalutamide *via* reducing the deposition of H3K27ac at the PSA promoter and inhibited the proliferation of PCa cells (144). More importantly, they showed that A-485 could inhibit tumor growth *in vivo*, using an AR-positive, patient-derived castration-resistant xenograft model, making it a promising candidate for clinical trials. In the same year, Jin et al. examined the effect of GNE-049, (a potent inhibitor of the CBP/p300 bromodomain) on histone acetylation and expression of AR target genes and found a significant decrease in H3K27ac at AR/p300 overlapping peaks, which inhibited AR-mediated transcription, although AR–p300 interaction or binding of the AR or p300 to the target gene promoter was not found to be inhibited by this compound (45). Interestingly, they found that GNE-049 could significantly inhibit the proliferation of AR-positive PCa cell lines and patient-derived tumor xenografts (PDX) models of PCa. Recently, CCS1477 was developed as a potent, selective, and orally bioavailable small-molecule inhibitor of the p300/CBP conserved bromodomain, and has been shown to inhibit cell proliferation in PCa cell lines, patient-derived xenografts, as well as in serial tumor biopsies acquired from an ongoing human phase I trial, by inhibiting AR regulated gene expression independent of c-MYC signaling (145). Preliminary clinical trials with CCS1477 demonstrated that combined administration of enzalutamide with CCS1477 enhances the efficacy of enzalutamide to further reduce tumor growth, compared to CCS1477 treatment alone. In addition, CCS1477 treatment has also been shown to modulate plasma KLK3 levels, expression of other AR target genes and CRPC biopsy biomarkers (145). The success of CCS1477 in ongoing clinical trials will encourage efforts to develop effective therapeutic compounds specifically for CRPC. Similarly, efforts have been made to target other lysine acetyl transferases to find better treatment options for PCa. In 2004, Chiao et al. determined the anticarcinogenic effects of PEITC-NAC, a major metabolite of PEITC, (phenethyl isothiocyanate, a natural compound derived from cruciferous vegetables), in the context of PCa and showed that it can significantly inhibit the growth of PC-3 xenografted tumors in immunodeficient mice (146). Several mechanisms have subsequently been proposed for the PCa preventive activity displayed by PEITC, including caspase-dependent apoptosis induction in TRMP-derived cells, through inhibition JAK-STAT3 signal-cascade activation in PCa cells, inhibition of IL6-induced AR transcriptional activity in LNCaP cells, by altering miRNA expression, reducing protein levels of MMP2 and MMP9 in PCa cells, and through inhibition of c-Myc expression and c-Myc regulated glycolysis in PCa cells (147–150). A study by Yu et al. highlighted that PEITC-mediated inhibition of AR-regulated transcriptional activity and growth of PCa cells was due to miR-17-mediated decrease in the expression of PCAF (151).

The fact that TIP60 can translocate and transactivate the AR in an androgen-independent manner indicates its importance in AR signaling and makes it an attractive target for both androgen-dependent and androgen-independent PCa therapy. Coffey et al., using HTS and targeted compound synthesis, identified NU9056 (1,2-bis(4-pyridyl)-ethan; an isothiazole), a highly-selective and potent inhibitor against TIP60 catalytic activity, and showed that NU9056 treatment could inhibit cell growth and colony formation in several PCa cells including androgen sensitive LNCaP and insensitive PC-3 cells (152). Furthermore, increased apoptosis and reduced PSA expression were observed in NU9056-treated cells, suggesting a therapeutic role for TIP60 inhibitors in the treatment of castrate-resistant PCa. Another compound, TH1834, was designed on the scaffold of PNT and acetyl CoA in search of an additional TIP60 inhibitor and interestingly, treatment with this compound in AR negative PCa cells induced DNA damage and cell death in treated cells (153). One of the challenges in using TIP60 as a therapeutic target is that TIP60 is an essential factor in mounting the DNA damage repair response and apoptosis in damaged cells, therefore, targeting TIP60 can make normal cells vulnerable to genotoxic stress and put them at risk of becoming cancerous. In addition to this, several other inhibitors have been reported against various KAT proteins, however, they have not yet been tested against PCa. For example, CoA-Ac-EEE4 was reported to be a highly potent and selective inhibitor of ARD1/hNaa10, with an IC_50_ value of 10.1 ± 1.4 μM, although this has not been tested against PCa (154).

In addition to synthesized chemical inhibitors, several compounds obtained from natural sources have exhibited the potential to inhibit acetyltransferase activities of many KAT proteins and have been associated with the prevention of cancer and other diseases (155). However, the mechanisms of action for many of the reported naturally derived KAT inhibitors are poorly understood and the off-target effects exhibited by many of these compounds preclude them from either undergoing clinical trials or becoming a major factor for their failures in clinical trials. For instance, garcinol, commonly found in the rind of the dried fruit of *Garcinia indica*, shows non-specific inhibitory activity against both p300 and PCAF, while anacardic acid extracted from the cashew nut shell, is a broad-spectrum KAT inhibitor that can non-selectively inhibit p300, PCAF, and TIP60 activity (155–159). The anti-prostate cancer properties of garcinol examined in human PCa cells and prostate tumor xenograft mouse models showed that garcinol decreased cell viability of treated cell lines and significantly reduced the tumor size by inducing apoptosis and inhibiting autophagy (160). Lee et al. found allspice hot water extract to be a potent inhibitor of p300 and CBP HAT activity and showed that allspice treatment significantly reduced AR acetylation and acetylation of histone H3 in the PSA promoter region, leading to reduced transcription of AR target genes and reduced growth of LNCaP cells (161). Despite showing promising effects in cell culture and xenograft models of prostate cancer, information on clinical trials of many of these KAT inhibitors is not available in the literature and requires intensive investigation to establish the safety and effectiveness of these KAT inhibitors before they are approved for clinical trials in humans.

## Conclusion

**Figure 3:**
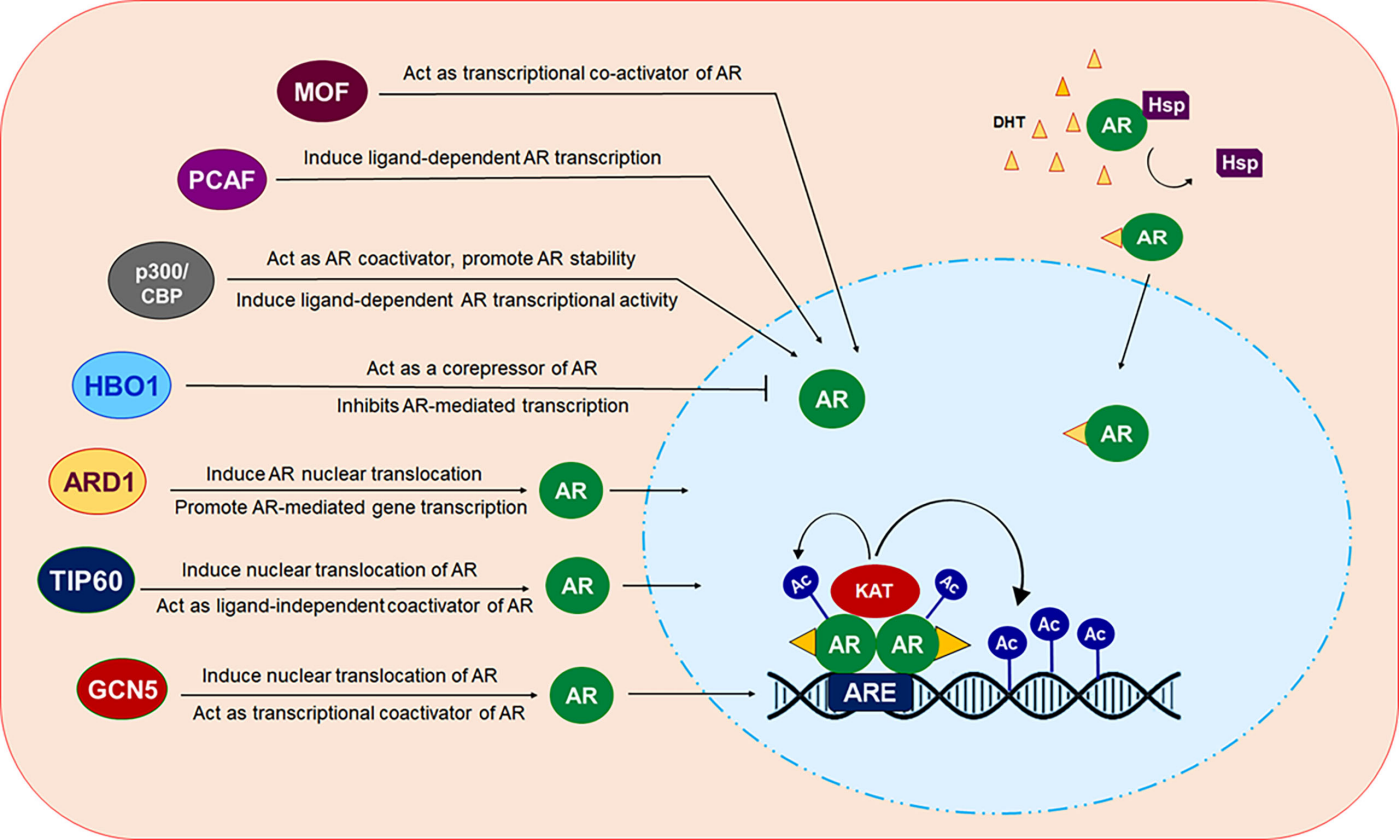
Modulation of AR by different KAT proteins. In presence of androgen ligands, AR which normally resides in the cytosol in a monomer form, translocated inside the nucleus. Ligand bound AR dimerize and binds at androgen response elements (AREs) in the target gene promoters/enhancers to modulate the transcription of targeted gene. KAT proteins ARD1, TIP60 and GCN5 can induce translocation of AR from cytosol into the nucleus. All of these KAT proteins, except HBO1 act as transcriptional coactivator of AR. HBO1 act as co-repressor of AR and inhibit AR signaling.

The future of CRPC treatment depends on alternative treatment strategies that requires identification of novel drug targets besides the AR which may overcome the health risk encountered by currently available drugs. In the last few decades, several studies have reported the contribution of deregulated expression and function of AR-related coregulators including various KATs, to aberrant AR activity during the onset of PCa development and progression (**Figure 3**). These findings have raised considerable interest in the use of targeting KATs as an alternative way to target AR signaling and facilitated the development of novel compounds that inhibit or restrict their activity. Despite demonstrating promising results in inhibiting prostate cancer cell growth in *in vitro* settings or in pre-clinical models of solid tumors, most of the KAT inhibitors, unfortunately, failed to reach the level of clinical trials for use in humans, in comparison to HDACs. A crucial factor that hinders the way for these agents to reach the clinical testing phase is the vital nature of KATs for growth and survival of normal cells. Moreover, various KATs catalyze acetylation of both histones and many non-histone proteins as their substrate, and thereby, suppressing their activity could significantly impact many biological processes and can have aggressive side effects. Most importantly, a KAT may act as an oncoprotein in one cancer and a tumor suppressor in another. PCAF plays an oncogenic role in breast cancer but acts as a tumor suppressor in PCa. In some cases, the structurally similar catalytic domain of KATs belonging to the same family may make development of selective inhibitors a difficult task to achieve. To overcome these problems, one approach could be to screen and identify agents that either inhibit or disrupt interaction of KAT with the AR by masking KAT domain involved in interaction with the AR instead of obstructing the catalytic function of these essential enzymes. Many KATs express and function in cell/tissue specific context, therefore, additional studies would help to identify and target specific KATs to control AR signaling, specifically in a tissue specific manner,without hindering AR activity in other tissues that may help to reduce undesired toxic effects.

Another approach could be to indirectly block KAT mediated acetylation of AR by blocking AR target sites acetylated by KAT proteins. The hinge region contains part of the NLS that regulates AR nuclear localization and most KATs target lysine residues within this hinge region, clearly showing the importance of the hinge region in modulating AR activity under both normal and pathological conditions. Indeed, like other functional domains of AR, several mutations in and around the hinge region have been reported in AIS as well as PCa patients, which impairs various aspects of AR function, including its intracellular dynamics, N/C interaction, hormone-binding efficiency, and recruitment of coactivators (16). In addition, several lysine acetyl transferases such as p300, P/CAF, and TIP60 have common acetylation sites located in the hinge region at residues K630, K632, and K633. Furthermore, compared to other functional domains, the poorly conserved nature of the hinge region can be exploited for the development of small peptides, monoclonal antibodies with epitopes in the hinge region, or chemical agents that could occupy the hinge region or mask acetylation sites located in this region, and can obstruct various KATs to acetylate AR to control KAT-mediated activation of AR. However, this would require detailed understanding of the role of the hinge region in regulating AR activity supported with molecular dynamics (MD) simulation and X-ray crystallography studies. Undoubtedly, there are still a number of challenges that stand in the way of successfully translating these findings into their application for clinical purposes and, of course, enough scientific evidence and information is needed to determine the safety and effectiveness of these drugs before clinical trials can be initiated.

## Author Contributions

Study concept and design: BJ and AG; Drafting of manuscript: BJ, AA, and AG. All authors contributed to the article and approved the submitted version.

## Funding

This work was supported by a grant from Indian Council of Medical Research (ICMR), India BMS/ADHOC/CMB/ 2015-0817/JUN-16/1 /UP/PVT to AG and DST grant (SR/WOS-A/LS/228/2017) to BJ. AA acknowledge financial support from the DST-INSPIRE fellowship (DST/INSPIRE Fellowship/2019/IF190432).

## Conflict of Interest

The authors declare that the research was conducted in the absence of any commercial or financial relationships that could be construed as a potential conflict of interest.

## Publisher’s Note

All claims expressed in this article are solely those of the authors and do not necessarily represent those of their affiliated organizations, or those of the publisher, the editors and the reviewers. Any product that may be evaluated in this article, or claim that may be made by its manufacturer, is not guaranteed or endorsed by the publisher.
